# Paid Domestic Work and Depressive Symptoms in Mexico: Results of a National Health Survey

**DOI:** 10.3390/ijerph21121566

**Published:** 2024-11-26

**Authors:** Germán Guerra, Amado D. Quezada-Sánchez, Claudine Burton-Jeangros, Arturo Júarez-García, Antoine Flahault, Nelly Salgado de Snyder

**Affiliations:** 1 Institute of Global Health, Faculty of Medicine, University of Geneva, CH-1202 Geneva, Switzerland; german.guerra@etu.unige.ch; 2 Global Health Program, Center for Health Systems Research, National Institute of Public Health, Cuernavaca 62100, Mexico; 3 Center for Evaluation and Surveys Research, National Institute of Public Health, Cuernavaca 62100, Mexico; 4 Institute of Sociological Research, Geneva School of Social Sciences, University of Geneva, CH-1212 Geneva, Switzerland; claudine.jeangros@unige.ch; 5 Center of Transdisciplinary Research in Psychology, Autonomous University of the State of Morelos, Cuernavaca 62209, Mexico; arturojuarezg@hotmail.com; 6 Latino Research Institute, College of Liberal Arts, The University of Texas at Austin, Austin, TX 78712, USA; nelly.salgado@austin.utexas.edu

**Keywords:** domestic work, depression, Mexico, domestic workers, informal work, depressive symptoms, mental health, occupational health

## Abstract

Paid domestic work (PDW) is an important source of employment for the global female workforce. It is frequently performed under precarious working conditions and occupational risks that are often associated with depressive symptoms (DSs). Although 10% (2.2 million) of Mexican working women are paid domestic workers (PDWs), their mental health has remained understudied. This article analyzes the occurrence and factors associated with DSs in a sample of Mexican workers segmented into six workforce groups, including PDWs. A three-stage statistical analysis was performed on national health survey data from Mexico (ENSANUT 2012): 1. Tabulation of inferential statistics; 2. Multiple logistic regression modeling of DSs; 3. Postestimation of DSs prevalence. Our findings suggest a context of social disadvantages based on gender, education, and labor market segregation that manifests in PDWs having the highest adjusted prevalence of DSs (14.1%, 95%CI = 9.7–18.4). Significant differences in prevalence were observed when compared with other workforce groups, such as formal employees. Among working women, after adjusting for relevant covariates, the odds of DSs were higher among PDWs as compared with formal employees (OR = 1.65, 95%CI = 1.04–2.61). Ongoing efforts for PDW formalization should be maintained in Mexico as an employment policy and mechanism to achieve PDWs’ social well-being and mental health.

## 1. Introduction

Paid domestic work (PDW) is an important source of employment for the global workforce. According to the International Labour Organization (ILO), paid domestic workers (PDWs) accounted for almost 5% of the global working population in 2021. About 75% of all 76 million PDWs worldwide are women, comprising 9% of the global working female population [1].

Common PDW activities include house cleaning, laundering, and meal preparation. Caregiving to children and the elderly is also frequent [2]. Since these activities are typically attributed to female gender roles, it is one of the few paid work options that socially disadvantaged women can access in developing countries [3,4]. Although men are not excluded from PDW, they perform mostly male role activities such as guarding or vehicle driving [2,5]. These demographic and occupational characteristics of PDW are instances of the class-based and gendered segregation of the global labor market [1].

Despite its vital role in reproducing daily life in households, PDW is a socially undervalued activity [6,7]. This results in PDWs being frequently overlooked as a workforce group worthy of focused well-being policies. Consequently, PDW has historically remained an informal low-paid job with precarious working conditions that is seldom regulated and frequently unsafe for physical and mental health [1,8].

In this adverse context, PDWs are chronically exposed to work-related stress and psychosocial risk factors, which are associated with common mental disorders (CMDs) such as depression or anxiety [9,10]. Some of these risks include performing activities during extended work shifts, with low control, high demand, and poor autonomy [11,12]. In addition, violence and abuse based on gender and class make PDWs particularly vulnerable to suffering from depression [13,14].

Research on PDWs and CMDs is scarce, particularly in low, lower-middle, and upper-middle-income countries (LLMUMICs). A systematic review of depressive symptoms (DSs) among these workers identified research gaps for increasing the evidence on their occupational mental health. Some of these gaps are the absence of research designs assessing causal associations between PDW and the onset of DSs or the scrutiny of factors beyond the workplace that act as stressors, such as work–family conflict and role strain. Furthermore, the review suggests that certain cultural and socio-economic factors—such as living in poverty, experiencing gender, ethnicity, and class based-discrimination, or residing in a country with ties to colonial past—act as drivers of female job placement in PDW. In turn, the interplay of those factors structures the working conditions and psychosocial risks—such as physical and verbal abuse, isolation, or irregular and extended hours of labor—which are associated with the occurrence of DSs. Finally, the review suggests that PDWs suffer from DSs more frequently than other workforce groups [15].

Some studies from Latin American countries seem to support such a claim. For instance, research conducted in the state of Salvador, Brazil, reported that PDWs had the highest adjusted-prevalence ratio of certain DSs, such as sadness or low concentration, compared with non-PDWs (2.22, 95%CI = 1.28–3.84) [14]. Also, another study in the same state estimated that PDWs had the highest prevalence of DSs (46.7%) compared to workers in other sectors, such as services (18.3%) [16]. Likewise, in Buenos Aires, Argentina, DSs among female PDWs were almost twice as prevalent than in non-PDWs (53.2% vs. 28%) [17].

In Mexico, despite its large PDWs population, studies on DSs are almost null. According to official estimates, there are 2.5 million PDWs in Mexico; 87.7% are women. PDWs represent 0.9% and 10.3% of the male and female workforce, respectively. Almost 97% of them are informal workers [5,18].

To the best of the authors’ knowledge, the only study analyzing DSs among Mexican PDWs was conducted by Soria-Trujano and Mayen-Aguilar and published in 2017 [19]. Using a non-probabilistic sample of 200 female PDWs residing in two metropolitan areas, the authors estimated the prevalence of DSs. They found that 39% of PDWs in Mexico City and 25% of those in Puebla had some level of depression (although the DSs difference between PDWs from each city was non-statistically significant). This study, however, did not adjust for covariates (i.e., age, education, marital status, number of children) that could confound the association between working in PDW and suffering from DSs. Furthermore, the study sample did not include other workers for comparison.

This paper aims to fill some gaps in the research of PDWs’ mental health. We analyze the presence of DSs in a sample of Mexican workers segmented into six workforce groups, including PDWs. We assess the association of DSs with relevant sociodemographic characteristics, working conditions, and socio-economic indicators. The objectives of this paper are: (1) to identify if PDWs are more affected by DSs than other workforce groups, as previous research suggests, and (2) to assess if the association between PDW and DSs is present after considering relevant socio-economic and demographic characteristics not previously analyzed among Mexican PDWs.

By pursuing these objectives, we test two hypotheses: (1) Mexican PDWs suffer from DSs more frequently than other workforce groups and (2) PDW remains associated with DSs after adjusting for other relevant factors related to developing these symptoms.

## 2. Materials and Methods

### 2.1. Data

We analyzed data from the National Health and Nutrition Survey of Mexico (ENSANUT), a representative population survey at the national, state, and rural/urban levels with a stratified and multistage probabilistic sampling design and variable periodicity of collection [20]. Data are available at the individual and household levels.

Although ENSANUT is not a source for employment statistics, nor is it focused on estimating work and employment conditions, it is one of the few sources available in the Mexican information system that includes data on mental health and morbidity and has limited information on employment status and occupation. Furthermore, vulnerable workforce groups can be identified according to some sociodemographic characteristics and working conditions. This study analyzed data collected in 2012 from 20,893 individuals. (See Supplementary Figure S1 for a detailed flowchart of dataset formation, including criteria selection of individuals).

It is important to acknowledge that more recent data from later rounds of ENSANUT are available. However, due to the lack of harmonization of questionnaires from the 2012 and later rounds, the information of items that allow for identifying workers in PDW activities is only available for 2012 (more detail is provided in Section 2.2).

### 2.2. Variables

The outcome variable in our study was the occurrence of DSs of clinical significance experienced within a seven-day lapse prior to the date of data collection. DSs were measured with a seven-item abbreviated version of the Center for Epidemiologic Studies Depression Scale (CESD-7). This scale has been validated for determining DSs in the Mexican population by Salinas et al. [21]. Following this study, we set a cut-off point at a ≥9 score (min-max range: 0 to 21) for the presence of DSs of clinical significance and coded the score into a dichotomous variable. It is worth noting that CESD-7 is not designed and should not be used as a basis for diagnosing depression but rather as a screening tool for clinically significant DSs.

We included three sets of predictor variables:

1. Sociodemographic: Age; sex; ethnicity; marital status; household headship; number of children under age 6; and number of children aged 6–11. Except for age and number of children, these are categorical variables. We chose the last three variables to approach sources of mental distress associated with DSs, such as work–family conflict. Household headship is a term that is technically and theoretically assigned to the household member whom the other members acknowledge as the main one responsible for decision making and access to economic resources. Consequently, this variable proxies the roles of breadwinning and decision making on household resources, while “number of children” approximates the role of childrearing [22]. Under the premise that fulfilling these roles depends on work and family balance [23], we included these variables to assess the relationship between work–family conflict and DSs in the studied sample.
2. Work and employment, including some working conditions as proxies of psychosocial risks at work: Workforce group (six categories of workers: formal, informal, PDWs, family-owned business, self-employees, and non-landowner farmworkers); extended work shift (more than 48 h weekly); and workplace violence. These variables are categorical. Extended work shift and workplace violence are dichotomous, indicating the presence or absence of these events.

Each level of the workforce group variable indicates the insertion of the working population into disadvantaged occupational categories (PDWs, family-owned business, self-employees, and farmworkers) or an important characteristic of the institutional arrangements of the Mexican labor market (formal or informal employment) [24,25]. Additional notes on the coding process of this variable are presented:

- The identification of PDWs in ENSANUT was not straightforward, since PDW is not explicitly included as an occupation. Nonetheless, the skip-logic sequence of the 2012 round questionnaire allows for classifying a subset of respondents as PDWs. They were individuals who initially declared not to have a job but answered positively when asked about obtaining a payment in exchange for activities typically conducted by PDWs within the seven-day reference period. This sequence of questions is consistent with one of the ILO’s approaches for estimating the number of PDWs using household surveys and census data (status in employment) [26]. This approach is useful when the data source lacks specific questions for classifying occupations, like ENSANUT. It is worth mentioning that ENSANUT’s household resident questionnaire from the 2012 round is the only version that includes the specific items (2.21 and 2.22) that allow for identifying PDWs under the status in employment approach. Items in more recent rounds omit some activities and include others that are not typically carried out by PDWs.
- Workforce groups were classified in two steps. First, respondents were grouped according to a set of labor market segmentation criteria based on the integrative classification of informal employment by Hussmanns [27]. Formal workers (labeled as “formal employees”) were those that declared having met two conditions: (a) to have job-related access to healthcare and (b) to have an employment-based retirement plan. In the second step, uncategorized respondents were classified into one of five mutually exclusive categories of informal workers based on their regular activities: PDWs, family-owned business, self-employees, other informal (waged) employees, and non-landowner farmworkers. This categorization of informal workers into different occupational categories is essential in our analysis, as it allows for the identification of DSs and socio-economic disparities within informal work. It also allows for testing whether PDWs is the group most affected by DSs among other informal workforce groups, as previous research suggests [14,15,16,17,28]. (For a detailed flowchart describing how the workforce group variable was generated and how PDWs were identified and classified within the whole dataset formation process, please see “Figure S1” in Supplementary Materials).

For approaching the psychosocial risks associated with DSs, extended work shift and workplace violence variables were included. These variables are particularly relevant for analyzing the exposure to distressful conditions among PDWs [15]. Although these are not the only psychosocial risk factors variables associated with DSs, they were the only ones we could identify with ENSANUT data. Moreover, although ENSANUT does not include validated tools for assessing psychosocial risks at work, it contains questions about experiences of violence (i.e., physical, verbal, sexual) within the last 12-month period and place of occurrence (at work, home, school, or other). We included these questions to probe workplace violence as psychosocial risk in our study.

3. Socio-economic: Years of schooling; residence area; and housing environment index. Residence area is a categorical dichotomous variable. Based on the population criterion used by the National Population Council of Mexico [29], localities with less than 2500 inhabitants were classified as rural. More populated areas were categorized as urban. Years of schooling and housing environment index are numerical variables. The former was split into several categories equivalent to educational stages in Mexico (zero years or no formal education; six years or primary; nine years or secondary; up to 15 years or middle-high or technical; and years ≥16 or professional). The housing environment index (HEI) is a proxy measure of socio-economic status [30] based on 14 dwelling characteristics, such as access to water and electricity (2), floor and ceiling construction materials (2), and possessions of personal or movable goods (10). The HEI was obtained by a principal component analysis (see Supplementary Materials “Table S1”). The percentage of variance explained by the first principal component (PC) was 27.5%. The correlation coefficients between the household characteristics and the first PC ranged from 0.62 to 0.25, with a mean of 0.51. The HEI was standardized (M = 0; S.D. = 1) considering the key elements of the survey design: stratification, primary sampling units, and sampling weights.

### 2.3. Statistical Analysis

Our analysis was conducted in three steps: (1) crosstabulation of variables across the working population sample with inferential statistics; (2) multiple logistic regression modeling of DSs; and (3) postestimation of DSs prevalence.

The crosstabulation included all previously listed variables disaggregated by workforce groups. Categorical variables were expressed as survey-weighted percentages. Mean age and HEI (continuous variables) were shown as raw and standardized scores, respectively. Ninety-five percent confidence intervals were calculated for all relative frequencies. Chi-square tests for contingency tables and two-way ANOVAs with F-tests were run on the tabulates of categorical and continuous variables, respectively.

We fitted a multiple logistic regression model for DSs adjusted for selected sociodemographic, work and employment, and socio-economic covariates. Two variables were omitted from the model due to low case count (workplace violence) or collinearity (number of children aged 6–11). Since PDW is mostly a feminized occupation, the model was run twice, first on the whole sample and then on women only. This allowed us to focus solely on DSs among working women and compare how these symptoms diverge with the whole working population. We expressed variable coefficients as odds ratios with 95% confidence intervals.

Finally, based on the adjusted predictions from the logistic model, a postestimation of DSs prevalence for each workforce group was calculated through predictive margins [31]. We set the significance level at 0.05 for all statistical tests.

We required completeness of all relevant covariates for each individual to conduct our analysis. After selecting the working population sample, we identified and discarded those observations with incomplete data on covariates, following a listwise deletion method. The eliminated observations accounted for 3.03% of all working population sample.

All analyses were performed considering the key elements of the survey design: stratification, primary sampling units, and sampling weights. Standard errors were obtained through the linearization method [32]. Database management and statistical analysis were performed with Stata statistical software package version 18.

## 3. Results

Table 1 summarizes the prevalence of DSs, the sociodemographic characteristics, and the working conditions of the studied sample, disaggregated by workforce groups.

The prevalence of clinically significant DSs varied according to workforce group (*p* < 0.001). The highest prevalence of DSs was observed in PDW (23.7%, 95%CI = 17.1–30.4) and family-owned business (17.7%, 95%CI = 12.3–23.1). Formal employees had the lowest DSs prevalence (7.4%, 95%CI = 6.2–8.6).

The sociodemographic and socio-economic indicators provide an overview of the living conditions of this Mexican workforce sample. When compared to the total working population, PDWs are characterized by fewer years of schooling (the second highest proportion of no formal education after farmworkers: 30%, 95%CI = 22.4–37.6) and a lower proportion of household headship (29.8%, 95%CI = 23–36.7 vs. 53.5%, 95%CI = 52.4–54.7 in the general working population). Also, PDW is the most feminized workforce group in the sample, with more than twice the proportion of working females compared to the total workforce population (87.5%, 95%CI = 81.1–94 vs. 40.5%, 95%CI = 39.4–41.5). Finally, the house environment index of PDWs is below the general working population (−0.3, 95%CI = −0.5–0.1) and formal workers (0.4, 95%CI = 0.4–0.5).

Regarding adverse working conditions, almost one-third of the total working population experienced a work shift of more than 48 h a week. However, among PDWs, this proportion was the lowest compared to the other groups (7.4%, 95%CI = 3.9–11). Lastly, workplace violence was rarely declared in this sample.

Table 2 shows the estimates of the logistic regression as adjusted odds ratio (OR). PDWs, self-employees, and informal employees showed increased odds of DSs compared to formal employees. The highest odds increase for DSs was observed in PDWs (1.61, 95%CI = 1.05–2.48). These results were similar among working women. In the female population, only farmworkers had lower odds of DSs compared to formal employees (0.46, 95%CI = 0.22–0.98).

The sociodemographic characteristics associated with increased odds of DSs were female sex (OR = 3.26, 95%CI = 2.66–3.99) and age (OR = 1.12, 95%CI = 1.03–1.23). Among women, being the head of household was associated with higher odds of DSs (OR = 1.42, 95%CI = 1.13–1.79). Conversely, having children aged 0–5 years old was associated with lower odds of DSs across the whole sample of workers and among working women (0.82, 95%CI = 0.69–0.97 and 0.82, 95%CI = 0.68–0.99, respectively). Socio-economic variables associated with lower odds of DSs were years of schooling (OR = 0.94, 95%CI = 0.92–0.96 for both sexes; OR = 0.95, 95%CI = 0.92–0.97 for women) and HEI (OR = 0.91, 95%CI = 0.84–0.98). However, association of HEI with DSs was not significant in women. Ethnicity, marital status, and extended work shift were not statistically significantly associated with DSs.

**Figure 1:**
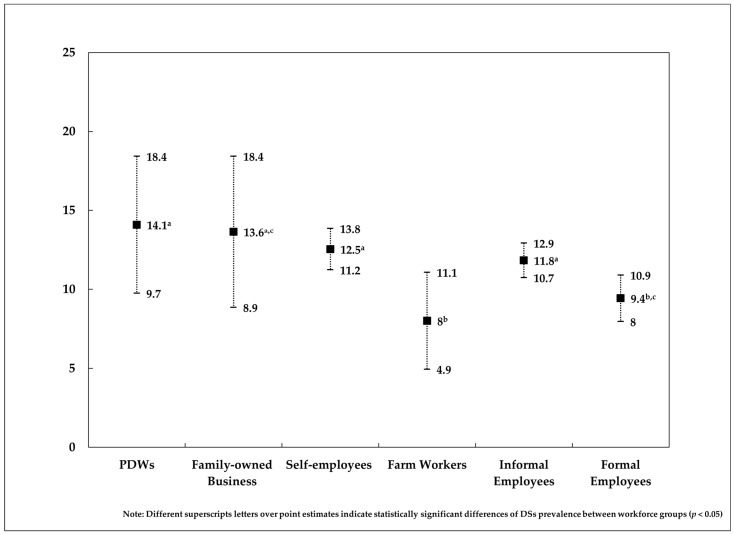
Adjusted prevalence of depressive symptoms by workforce group. Both sexes. Mexico. 2012.

Figure 1 displays the adjusted prevalence of DSs with 95% confidence intervals for each workforce group based on the model estimates. After adjusting for all the model covariates, the point estimate prevalence of DSs is still the highest in PDWs (compare with Table 1), but it was closer to the family-owned business group. Furthermore, despite the wide confidence intervals and overlap of DSs prevalence between some workforce groups, statistically significant differences in DSs prevalence between PDWs were observed when compared with formal employees (+4.65%; 95%CI = 0.01–9.29) and farmworkers (+6.1%; 95%CI = 0.89–11.26). These differences are referenced by superscript letters next to the DSs prevalence value for each workforce group (see Figure 1 footnote. All pairwise contrasts of DSs prevalence between workforce groups are available in Supplementary Materials “Tables S2 and S3”).

## 4. Discussion

In this contribution, we aimed to strengthen the evidence of an adverse mental health outcome and its social determinants in a vulnerable group of the Mexican labor market. We built our study upon the scarce research on DSs among PDWs in Mexico [19,33] and formulated two hypotheses. Firstly, we tested if these workers are more frequently affected by DSs than other workforce groups. Then, we assessed if PDW was associated with DSs when other socio-economic and sociodemographic factors were accounted for in the relationship. To the best of the authors’ knowledge, no previously published studies on occupational mental health in Mexico have analyzed and compared DSs among PDWs and other workforce groups using data from a national health survey. In the following subsections, we discuss our results in the light of the hypotheses and the contribution this study makes to the current state of research on PDW in Mexico. Lastly, we close the discussion section with policy recommendations.

### 4.1. Hypothesis 1: Mexican PDWs Suffer from DSs More Frequently than Other Workforce Groups

Unadjusted prevalence of DSs depict a clear poor mental health gradient according to occupational categories, where PDWs suffer more frequently from these symptoms than the rest of the analyzed workforce groups and the general working population (Table 1). This result provides further evidence to the hypothesis of PDW being one of the most hazardous jobs for DSs when compared to other disadvantaged occupations [15]. However, when prevalence is adjusted for all other relevant variables, the gradient-like trend loses its slope, suggesting similar levels of DSs across the Mexican workforce (Figure 1). Particularly for PDWs, differences in DSs prevalence are significant only when compared with formal employees and farmworkers. Three observations can be derived from these results.

Firstly, the similar adjusted prevalence of DSs between PDWs and other workforce groups could be explained by measurement or sampling limitations, resulting in larger confidence intervals for the estimates of workforce groups with fewer respondents (PDWs, family-owned business, and farmworkers). Unless improved measure instruments or sampling designs that allow for more precise estimations of underrepresented workforce groups are applied in the future, these adjusted prevalences can be provisionally considered as the most reliable.

Secondly, beyond plausible explanations of statistical and data collection order, the similar adjusted DSs prevalence between PDWs and other vulnerable workforce groups (family-owned business, informal employees, and self-employees) can be explained by broader societal contexts and macroeconomic determinants that structurally pervade the Mexican labor market and affect its workforce’s mental health. Arguably, the most salient of these determinants is the persistent segmentation of the labor market into formal and informal sectors, which is also present in other Latin American countries, and is found to be associated with adverse health outcomes [34]. Likewise, as a consequence of the diffusion of adverse working conditions [35,36] towards the generalization of job precariousness across the whole Mexican working population, also experienced in industrialized countries [37], the risk distribution of DSs between workforce groups might be gradually becoming homogeneous or stationary, affecting even the less vulnerable informal workers.

Finally, certain sociodemographic and socio-economic indicators signal a specific context of additive and interlocking social disadvantages affecting Mexican PDWs. For instance, after farmworkers, PDWs exhibited the lowest proportion of later stages of educational attainment (middle-high and professional). Also, they had the lowest percentage of household headship, indicating that the decision-making role in these workers is less common than that in other workforce groups. (Table 1). This context reflects the uneven distribution of well-being and job opportunities across the Mexican workforce, where the less valued, more feminized, and high-risk occupations are mostly performed by socially disadvantaged groups [38]. These adverse conditions, interwoven by the threads of gendered relationships that result in labor market segregation, can be considered the social determinants of ill mental health among PDWs. Under a health equity lens [39], acting on these determinants—by enhancing educational opportunities, improving living conditions, and promoting equitable gender job opportunities and relationships—could eventually lead to narrowing the DSs gap between PDWs and the less vulnerable formal workers.

### 4.2. Hypothesis 2: PDW Remains Associated with DSs After Adjusting for Other Relevant Factors Related to Developing These Symptoms

The most relevant finding of this study is that even after adjusting for socio-economic and sociodemographic characteristics, working in PDW was associated with DSs. Moreover, among female workers, the odds of DSs for PDWs remained higher when compared to formal employees and farmworkers (Table 2). These results support our second hypothesis and contribute to closing the evidence gap on PDWs’ mental health. Also, they appeal to explanations as to why this occupation associates with adverse mental health outcomes, particularly in the Mexican context, as we now examine.

#### 4.2.1. An Approximation to Psychosocial Risks and an Explanation from the Structural Determinants of Health

We attempted to proxy some psychosocial risk factors associated with DSs by including questions about violence occurring in the workplace and extended work shift. However, neither was workplace violence frequently reported in the sample nor extended work shift associated with DSs (Table 1 and Table 2). In fact, extended work shift was less prevalent among PDWs compared to other groups, arguably because many PDWs are employed under a variable demand and daily wage basis, leading to fewer working hours [5].

Nonetheless, these results should not be interpreted as workplace violence seldom occurring or long work shifts not frequently affecting the Mexican working population when the opposite has been documented elsewhere [40,41]. Rather, these findings point to the need for more research on other psychosocial risks, which has rarely been conducted in Mexico and other Latin American countries with large proportions of PDWs in their workforce. For instance, some studies on PDWs from Chile [42], Mexico [43], and Peru [44] systematically report high psychosocial risks of quantitative demands (Chile, México) and pace (Perú); low influence in job control (Chile, Mexico); and low social support and leadership, including feeling of belonging (Chile) and support from colleagues (Peru). Moreover, a study based on a national representative working population survey in Chile [45] identified that PDWs had the highest scores on a 0–100 scale on certain psychosocial risks, such as salary unsatisfaction (64.7 vs. 50.3) or the feeling of having a meaningless job (31.4 vs. 21.5) when compared to the mean of other 26 workforce groups.

Additionally, further research on psychosocial risks should aim to broaden its approach, as some scholars have suggested [43,46], for assessing the relationship between non-traditional forms of employment [47,48] and the mental health of underprivileged groups. One such approach is to examine the structural determinants of health inequities that link macroeconomic and social contexts, such as the labor market and societal values, with mental health and well-being in Mexico [49]. Under this perspective, and the premise by Rojas-García and Toledo [38], the inherent hardships experienced by informal workers could be amplified if the performed occupation is undervalued. Consequently, PDWs might experience the exposure, intensity, and impact of these adversities on their mental health more frequently than people employed in more socially valued jobs.

As a consequence of this amplification effect, PDWs must increase their income through additional activities (such as waste-picking or street vending) [38] in a context of work uncertainty [50]. Like job insecurity, a concept used for assessing the individual’s perceptions of workplace-related events that threaten job continuity [51], prolonged exposure to work uncertainty is a factor associated with DSs [48]. However, unlike job insecurity, work uncertainty can occur and be caused by events beyond the workplace, such as labor market structure and policies [50] and can be joined by another aspect of precariousness: involuntary work flexibility [52,53]. In tandem, these adverse working conditions configure what some authors have coined as “forced self-exploitation” [38,54], which physically and mentally exhausts PDWs and can result in DSs.

Arguably, the potential consequences of forced self-exploitation and social devaluation of PDW on mental health might reach further areas of PDWs’ well-being. One instance is the delayed emergence of PDWs’ “class consciousness”, which would enable them to act against historical injustice and intersectional discrimination. If this class consciousness has been slowly emerging, it is because the need of Mexican PDWs to procure their daily living conditions is greater than their collective organization. This can also explain the relatively slow pace of PDWs’ unionization in Mexico when compared to other Latin American countries [55].

Under this premise, a mutually involved link between mental health and class consciousness can be proposed, and a new hypothesis is formulated: workforce groups at greater risk of, or experiencing DSs, are hindered from raising awareness about their adverse conditions and from acting on the upstream determinants that structure health inequities, such as intersectional discrimination, unequal labor market opportunities, and exclusionary social policy.

#### 4.2.2. Work–Family Conflict and Role Strain: Unequal Distribution According to Employer–Employee Relationship

This study identified an association between female household headship and DSs. The association can be attributable to work–family conflict, as has been extensively documented in the literature [23,56,57,58,59], and the gendered role strain that results from juggling paid work with higher loads of unpaid domestic and care activities [60,61]. However, the impact of role strain on mental health affects women unequally, according to their job position in the labor market and social class [62]. Acknowledging these disparities is vital for examining further dynamics between CMDs and PDW.

Particularly relevant for the Mexican context or other LLMUMICs is the PDW employer–employee relationship. Not only does the PDW supply come from women, but also its demand [6]. This job relationship can be understood as an exchange between women, where the employer unloads her domestic and care work onto another woman. This role release not only waives the employer’s reproductive household activities but also increases her time to focus on productive work, reducing her role strain. It also allows her to engage in other activities, including those pursued by gender egalitarian agendas [63]. This exchange produces what some scholars have defined as “stratified reproduction” [64,65], and it could have different effects on mental health depending on whether the woman is on the demand or the supply side of the PDW. For PDWs, reducing the load of household activities off their employers entails an absorption of the mental distress that otherwise these employers would experience due to role strain. However, all this occurs at the expense of the worker’s mental health. As such, PDW has a buffering effect that cushions the employers against ill mental health [66,67,68] while at the same time taxes and depletes the resources and coping mechanisms that the worker requires to fend off adverse mental health outcomes, such as DSs.

### 4.3. Research on PDWs and Mental Health as a Neglected Yet Significant Public Health Concern in Mexico

The exiguous research on the mental health of Mexican PDWs contrasts with the numerous studies analyzing their adverse social context and working conditions [38,55,66,69]. These authors underscore the persistence of some elements of PDW’s colonial relations of production that explain why PDW is underpinned by ethnic, gender, and class discrimination.

Arguably, the most salient of these elements is its historical proximity to unpaid domestic labor. It explains the undervaluation of PDW as an economic activity and manifests in the state’s and employers’ hesitancy to regulate it as a formal employment relationship or PDWs’ reluctance to identify themselves as workers [38,55]. This context reflects the precarious working conditions, abuse, and labor rights violations that PDWs experience even today, exposing them to ill mental health and the development of DSs [64,69,70]. Given this context, PDWs’ occupational mental health should be deemed a pertinent research topic for Mexican scholars. Nonetheless, it has caught very little attention, and three reasons can explain such disinterest.

Firstly, due to methodological and ethical concerns, data collection on vulnerable groups is a challenging endeavor [39,71,72]. Specifically on Mexican PDWs, some authors have acknowledged certain hindrances to obtain reliable data for unbiased estimations of PDW’s health status, such as data collection in private households or PDWs’ availability for completing survey questionnaires [19,33].

Secondly, the availability of health and occupational information in single data sources is infrequent in the Mexican survey system. This results in limitations for linking health and occupational data from diverse sources. Since a specific survey on occupational health is yet to be conducted in Mexico [73], researchers and policymakers rely on existent health data sources with dissimilar information on work and employment. This is also the case of our study.

Thirdly, due to the increase in labor market participation of Mexican women in jobs other than PDW, research interests have shifted to study female occupational health in blue- or white-collar jobs and male-dominated areas [74]. The decline of women’s participation in the Mexican labor market as PDWs coincides with the decrease in published studies on PDW, particularly in the social sciences [33,63,75]. In 1910, almost half of the working women were PDWs. In 1930, they accounted for 42.3%. During the 1970s, only a quarter of the female workforce were employed in PDW. This rate steadily declined to 9%–10% in the 1990s, corresponding with current levels in the 2020s [5,66].

The research disinterest and societal context explain why, only up until very recently, Mexican PDWs have been considered a vulnerable group worthy of social security or well-being policies. Current efforts for improving PDW’s working conditions include the ratification of the ILO’s Convention 189 on decent work for domestic workers (C189) and labor law reforms for PDW formalization in 2019 [76,77]. Nevertheless, this progress could be improved if it is joined with sound evidence derived from reliable data and quality research on PDWs’ occupational mental health. This study takes the first step towards such a direction.

### 4.4. Policy Recommendation

The most critical policy recommendation that can be drawn from our results is to strengthen the ongoing efforts to improve Mexican PDWs’ well-being through employment formalization. Instances of this progress include the ratification of ILO’s C189 in 2019 and the mandatory social security affiliation of domestic workers by their employers. However, there is still a lengthy way to go. Up to May 2022, only 2% of the targeted population was effectively affiliated with the program (47,000 out of 2.3 million; own calculations based on official statistics [78]). Hopefully, the findings from this study will provide health and employment policymakers with further evidence of the potential effects of formalization on preventing adverse mental health outcomes. This study could also encourage them to sustain efforts to formalize other vulnerable workforce groups in Mexico besides PDWs.

### 4.5. Limitations

Due to the data type and design of the study, our results and interpretations are constrained by the boundaries of the social causation–social drift epidemiological hypotheses [36,79,80]. Therefore, we cannot ascertain whether the subjects in our sample suffered from pre-existent DSs that led them to lose their jobs and subsequently start employment as PDWs (supporting the social drift hypothesis) or rather their low socio-economic context narrowed their employment options to the most precarious jobs, exposing them to experience DSs [48] (supporting the social causation hypothesis).

In addition, although our estimates were obtained through a validated scale for assessing clinically significant DSs among the Mexican population, it must be stressed that a positive response is not indicative of a diagnosis of major depression. Moreover, the CESD-7’s seven-day reference period of collection can be considered a short timeframe leading to underestimation of DSs prevalence if compared with longer reference periods, such as those seeking to estimate the 12-month or lifetime DSs prevalence commonly set in cohort studies [81].

Another limitation relates to the data source. As mentioned, ENSANUT is neither a labor statistics nor occupational health survey. This situation limits the capacity to accurately characterize the working population in the same way as a specialized survey on employment would achieve. This is reflected in the reduced number of individuals classified as PDWs compared to other groups in this study, as well as to the numbers reported in the official statistics and literature on Mexican PDWs [5,66]. Therefore, we acknowledge that our results on PDWs’ DSs could be either under- or overestimated, and until this study is reproduced in a sample with a larger number of PDWs, we cannot determine the extent of this under- or overestimation. Moreover, given the elapsed time since the collection date of this survey (2012), our findings may not accurately reflect the current situation. We are compelled to point out these limitations not only because of due methodological rigor but also to illustrate previous findings and support the claims by other authors regarding the persistent obstacles in PDWs’ data collection in Mexico [33]. Until a specific occupational health survey becomes available in Mexico, ENSANUT could be further used to estimate DSs or other health issues in the Mexican workforce.

Despite these limitations, we consider our study to be a significant advancement and a starting point for future research on the mental health of PDWs and other vulnerable workforce groups. Subsequent studies should consider designs and methods that better approximate the causal nexus between PDW and DSs. Also, our findings underscore the need for improving health and occupational data availability and harmonization in Mexico to aid the generation of evidence-based mental health policies tailored to neglected workforce groups.

## 5. Conclusions

The results from our study make a substantial contribution to the evidence in Mexico on the relationship between disadvantaged occupations, such as PDW, and adverse mental health outcomes, like DSs.

The first hypothesis of our study—PDWs are more affected by DSs than other workforce groups—could only be partially backed up by our analysis, possibly due to the reduced number of PDWs in the sample or similar risks for DSs among vulnerable workforce groups. Nevertheless, significant differences in DSs prevalence between PDWs and formal employees were observed, suggesting that the former suffer from DSs more frequently than the latter. More precise estimates in the future require the inclusion and refinement of measuring methods and sampling designs for identifying PDWs in Mexican health surveys.

The second hypothesis—PDW is associated with DSs after other relevant factors are considered—was supported by our analysis. For working women, being employed in PDW resulted in the highest increase in the odds of DSs compared to women in formal employment, after adjusting for other covariates. For explaining this association, our study proposes broadening the scope of common analysis frameworks, such as psychosocial risks, by focusing on several context-specific societal factors of the Mexican labor market. Thus, work uncertainty, involuntary work flexibility, forced self-exploitation, and gender-based labor market inequities are some guiding concepts to include in future research aimed at understanding the relationship between PDW and DSs in Mexico and other countries with large populations of PDWs.

Current efforts for PDW formalization should be further maintained and encouraged as a labor market policy in Mexico. Alongside, existing organized groups should promote occupational mental health and raise awareness for collective action toward improving PDWs’ working conditions. After all, there can be no social change without mental health.

## Figures and Tables

**Table 1:** Sociodemographic characteristics, working conditions, and depressive symptoms by workforce groups. Mexico 2012.

	Total	Paid Domestic Workers	Family-Owned Business	Self-Employees	Farmworkers	Informal Employees	Formal Employees	*p*-Value ^b^
Sample size	20,893	328	536	6541	432	7671	5385	––
**Population Size** (Weighted; in thousands) ^a^	33,837	439	823	10,164	425	13,247	8739	––
**Sex** (Percent; 95% CI)								
*Female*	40.5 (39.4–41.5)	87.5 (81.1–94)	69.4 (63.4–75.5)	41.5 (39.5–43.6)	40.7 (33.6–47.7)	38.2 (36.6–39.9)	37.5 (35.3–39.7)	<0.001
*Male*	59.5 (58.5–60.6)	12.5 (6–18.9)	30.6 (24.5–36.6)	58.5 (56.4–60.5)	59.3 (52.3–66.4)	61.8 (60.1–63.4)	62.5 (60.3–64.7)	
**Mean Age** (CI)	39.5 (39.2–39.8)	42.2 (39.8–44.6)	40.7 (38.2–43.2)	44.2 (43.7–44.8)	46.2 (44–48.4)	36.4 (36–36.9)	37.9 (37.4–38.5)	<0.001
**Ethnicity** (Percent; 95% CI)								
*Indigenous*	19.3 (18.2–20.5)	19.8 (14.5–25.2)	26.8 (20.9–32.7)	23.1 (21.1–25.1)	44.4 (36.8–52.1)	18.8 (17.2–20.3)	13.8 (12.2–15.4)	<0.001
*Non-indigenous*	80.7 (79.5–81.8)	80.2 (74.8–85.5)	73.2 (67.3–79.1)	76.9 (74.9–78.9)	55.6 (47.9–63.2)	81.2 (79.7–82.8)	86.2 (84.6–87.8)	
**Years of Schooling** **(Stage Equivalent)** (Percent; 95% CI)								
*0 (No formal education)*	13.9 (13.2–14.7)	30 (22.4–37.6)	19.5 (14.6–24.5)	22.4 (20.8–23.9)	51.9 (45.7–58.2)	12.4 (11.3–13.5)	3.2 (2.6–3.8)	<0.001
*6 (Primary)*	19.1 (18.2–20.1)	28.2 (20.8–35.6)	19.4 (14.7–24)	23.4 (21.9–25)	22.6 (17.7–27.6)	21.3 (19.7–22.8)	10.3 (9–11.7)	
*9 (Secondary)*	30.1 (28.9–31.2)	30.4 (22.4–38.5)	21.6 (16–27.3)	28.8 (26.8–30.7)	19 (14–24)	32.9 (31.1–34.8)	28.7 (26.6–30.7)	
*13 (Middle-High, Technical)*	20.8 (19.8–21.8)	8.6 (3.2–13.9)	26.7 (17.3–36.2)	16.1 (14.6–17.6)	5.8 (1.6–10)	20.3 (18.7–21.9)	27.9 (26–29.8)	
*16 (Professional)*	16 (15–17.1)	2.8 (0.5–5)	12.8 (6.8–18.7)	9.3 (7.9–10.7)	0.6 (0–1.4)	13.2 (11.8–14.5)	29.9 (27.8–32.1)	
**Marital Status** (Percent; 95% CI)								
*Union*	67.7 (66.6–68.9)	68 (61–75)	67.5 (57.9–76.8)	71.3 (69.5–73.1)	75.9 (69.9–81.9)	62.6 (60.8–64.4)	71 (68.8–73.1)	<0.001
*Single*	32.3 (31.1–33.4)	32 (25–39)	32.6 (23.2–42.1)	28.7 (26.9–30.5)	24.1 (18.1–30.1)	37.4 (35.6–39.2)	29 (26.9–31.2)	
**Household Headship** (Percent; 95% CI)	53.5 (52.4–54.7)	29.8 (23–36.7)	24.6 (18.7–30.5)	59.4 (57.4–61.3)	52.2 (45.1–59.2)	50.1 (48.4–51.8)	55.9 (53.4–58.3)	<0.001
**Number of Children Under Age 6** (Percent; 95% CI)								
*0*	71.2 (70.2–72.3)	62.8 (54.8–70.9)	72.5 (66.1–78.9)	74.1 (72.2–76)	72.6 (66.6–78.5)	69.5 (68–71.1)	70.8 (68.9–72.6)	0.001
*1 or over*	28.8 (27.7–29.8)	37.2 (29.1–45.2)	27.5 (21.1–34.9)	25.9 (24–27.8)	27.4 (21.5–33.4)	30.5 (28.9–32)	29.2 (27.4–31.1)	
**Number of Children Aged 6 to 11** (Percent; 95% CI)								
*0*	72 (70.9–73.1)	66.1 (58.9–73.4)	72.7 (66.4–79)	72.4 (70.5–74.4)	70.4 (64.2–76.5)	71.7 (70.1–73.2)	72.3 (70.3–74.3)	0.697
*1 or over*	28 (26.9–29.1)	33.9 (26.6–41.1)	27.3 (21–33.6)	27.6 (25.6–29.5)	29.6 (23.5–35.8)	28.3 (26.8–29.9)	27.7 (25.7–29.7)	
**Housing Environment Index** (Standardized)	--	−0.3 (−0.5–−0.1)	0 (−0.2–0.2)	−0.2 (−0.2–−0.1)	−1.3 (−1.5–−1.2)	−0.1 (−0.2–−0.1)	0.4 (0.4–0.5)	<0.001
**Residence Area** (Percent; 95% CI)								
*Urban*	85.4 (84.6–86.3)	80.8 (75.2–86.3)	75.9 (70.6–81.2)	80.8 (79.4–82.3)	20.6 (14.7–26.5)	87.1 (86–88.2)	92.6 (91–94.2)	<0.001
*Rural*	14.6 (13.7–15.4)	19.2 (13.7–24.8)	24.1 (18.8–29.4)	19.2 (17.7–20.6)	79.4 (73.5–85.3)	12.9 (11.8–14)	7.4 (5.8–9)	
**Extended Work Shift** **(>48 h weekly)** (Percent; 95% CI)	31.9 (30.8–32.9)	7.4 (3.9–11)	23.8 (18.2–29.5)	33.9 (32.1–35.8)	22.6 (16.5–28.7)	35.5 (33.7–37.2)	26.4 (24.5–28.4)	<0.001
**Workplace Violence** (Percent; 95% CI)	0.4 (0.2–0.5)	2.3 (2.1–6.6)	0 (0.0–0.0)	0.3 (0.1–0.4)	0 (0.0–0.0)	0.4 (0.1–0.7)	0.4 (0.2–0.6)	0.099
**Clinically Relevant DSs** (Percent; 95% CI)	11.6 (10.9–12.3)	23.7 (17.1–30.4)	17.7 (12.3–23.1)	14.5 (13–15.9)	12.1 (8–16.2)	11.4 (10.3–12.5)	7.4 (6.2–8.6)	<0.001

^a^ Estimates calculated over weighted sample. ^b^ Chi-square tests for contingency tables and two-way ANOVAs with F tests were run on categorical and continuous variables, respectively.

**Table 2:** Work and employment, sociodemographic, and socio-economic factors associated with depressive symptoms of clinical significance in the Mexican workforce. Mexico 2012.

	Total (N = 20,893)		Women (N = 9439)	
	Odds Ratio	95% CI	Odds Ratio	95% CI
**Work and employment**				
**Workforce group** (ref. Formal Employees)				
*Paid Domestic Workers*	1.61 *	1.05–2.48	1.65 *	1.04–2.61
*Family-owned Business*	1.55	0.97–2.48	1.32	0.83–2.08
*Self-employees*	1.40 **	1.10–1.77	1.41 *	1.05–1.9
*Farmworkers*	0.83	0.51–1.34	0.46 *	0.22–0.98
*Informal Employees*	1.31 **	1.07–1.60	1.33 *	1.03–1.72
**Work Shift** (ref. Regular (≤48 h))				
*Extended*	1.02	0.87–1.19	1.04	0.85–1.28
**Sociodemographic**				
**Sex** (ref. Male)				
*Female*	3.26 ***	2.66–3.99	--	--
**Age ^a^**	1.12 **	1.03–1.23	1.09	0.99–1.21
**Ethnicity** (ref. Non-Indigenous)				
*Indigenous*	1.04	0.87–1.24	1.00	0.80–1.24
**Marital Status** (ref. In Union)				
*Single*	1.07	0.89–1.28	0.87	0.71–1.07
**Household Headship** (ref. member not head of household)				
*Head of Household*	1.20	1.00–1.43	1.42 **	1.13–1.79
**Number of Children Under Age 6** (ref. No children under age 6)				
*No. of children under age 6 ≥ 1*	0.82 *	0.69–0.97	0.82 *	0.68–0.99
**Socio-Economic**				
**Years of Schooling**	0.94 ***	0.92–0.96	0.95 ***	0.92–0.97
**Housing Environment Index**	0.91 *	0.84–0.98	0.96	0.87–1.06
**Residence Area** (ref. Rural)				
*Urban*	1.02	0.86–1.21	0.95	0.77–1.18

* *p* < 0.05; ** *p* < 0.01; *** *p* < 0.001^; a^ Standardized, S.D. = 12.88.

## Data Availability

ENSANUT’s data is publicly available in the National Institute of Public Health’s ENSANUT repository at: https://ensanut.insp.mx/ (accessed on 13 November 2024).

## Supplementary Materials

The following supporting information can be downloaded at: https://www.mdpi.com/article/10.3390/ijerph21121566/s1, Table S1: Household characteristics and first principal component (pc1) correlation coefficients and score weights. Table S2: Predictive margins (adjusted depressive symptoms prevalence) by workforce group. Table S3: Pairwise contrasts of adjusted depressive symptoms prevalence. Figure S1: Flowchart of sample dataset formation.
