# Supplementary material for: Paid Domestic Work and Depressive Symptoms in Mexico: Results of a National Health Survey
Source: Int J Environ Res Public Health. 2024 Nov 26;21(12):1566. doi: 10.3390/ijerph21121566 (PMC11675248; doi:10.3390/ijerph21121566)
Supplement: Supplementary file 1 [file ijerph-21-01566-s001.zip › Figure_S1_Flowchart_Dataset_Formation.pdf]

Figure S1. Flowchart of sample dataset formation

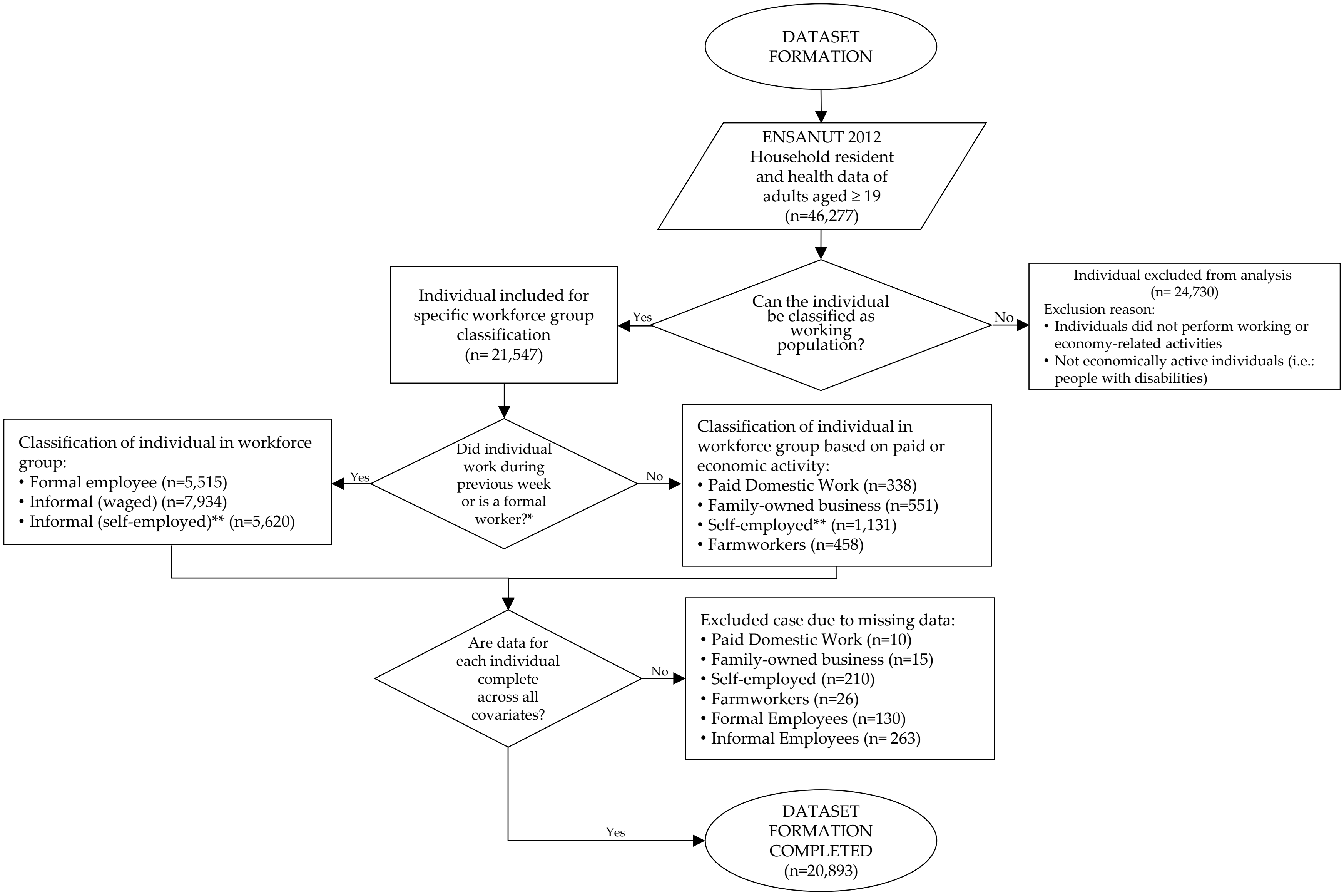

NOTES:

*Household questionnaire items on employment and occupational status within the reference period: "h221", "h222" and "h223". Number codes for item h221 included: 1 through 6 and 8. Number codes for item h222 included: 1 through 5. Number codes for item h223 included: 1 and 5. Household questionnaire items on formal employment: "h224a" and "h224e". The complete ENSANUT 2012's household data questionnaire is available at: https://ensanut.insp.mx/encuestas/ensanut2012/descargas.php (access date: November 6, 2024).

**Informal self-employed individuals that declared having worked within the reference period (n= 5,583) and those that worked as self-employees but declared not working within the reference period (n= 1,125) were assigned to the single workforce group "Self-employees".
