# Supplementary material for: Paid Domestic Work and Depressive Symptoms in Mexico: Results of a National Health Survey
Source: Int J Environ Res Public Health. 2024 Nov 26;21(12):1566. doi: 10.3390/ijerph21121566 (PMC11675248; doi:10.3390/ijerph21121566)
Supplement: Supplementary file 1 [file ijerph-21-01566-s001.zip › TableS2_S3. Pairwise Contrast of Adjusted DS prevalence.pdf]

Table S2. Predictive margins (adjusted depressive symptoms prevalence) by workforce group

Workforce Group	Prevalence (95% C.I.)
Paid Domestic Work	14.1 (9.7-18.4)
Family-owned Business	13.6 (8.9-18.4)
Self-employment	12.5 (11.2-13.8)
Farm Workers	8 (4.9-11.1)
Formal Employees	9.4 (8-10.9)
Informal Employees	11.8 (10.7-12.9)

Table S3. Pairwise contrasts of adjusted depressive symptoms prevalence

Workforce Groups	Contrast (95% C.I.)+	P-value
Paid Domestic Work vs Family-owned Business	0.44 (-5.83-6.72)	0.89
Paid Domestic Work vs Self-employment	1.55 (-3.00-6.09)	0.505
Paid Domestic Work vs Farm Workers	6.08 (0.89-11.26)	0.022
Paid Domestic Work vs Formal Employees	4.65 (0.01-9.29)	0.049
Paid Domestic Work vs Informal Employees	2.26 (-2.21-6.72)	0.322
Family-owned Business vs Self-employment	1.10 (-3.79-6.00)	0.659
Family-owned Business vs Farm Workers	5.63 (0.11-11.16)	0.046
Family-owned Business vs Formal Employees	4.21 (-0.84-9.26)	0.102
Family-owned Business vs Informal Employees	1.81 (-3.12-6.75)	0.471
Self-employment vs Farm Workers	4.53 (1.39-7.67)	0.005
Self-employment vs Formal Employees	3.11 (0.99-5.23)	0.004
Self-employment vs Informal Employees	0.71 (-0.95-2.36)	0.4
Formal Employees vs Farm Workers	1.42 (-2.05-4.89)	0.422
Informal Employees vs Farm Workers	3.82 (0.55-7.09)	0.022
Informal Employees vs Formal Employees	2.40 (0.64-4.16)	0.008

+For minuend and subtrahend of differences, refer to specific DSs prevalence in Table S2. Results may vary due to decimal rounding.
