# Supplementary material for: Paid Domestic Work and Depressive Symptoms in Mexico: Results of a National Health Survey
Source: Int J Environ Res Public Health. 2024 Nov 26;21(12):1566. doi: 10.3390/ijerph21121566 (PMC11675248; doi:10.3390/ijerph21121566)
Supplement: Supplementary file 1 [file ijerph-21-01566-s001.zip › TableS1. Household characteristics and first PC_4-Nov-24.pdf]

Table S1. Household characteristics and first principal component (pc1) correlation coefficients and score weights

Household characteristic	Correlation with PC 1	Score weights
Microwave[+]	0.6154	0.1599
Refrigerator[+]	0.6148	0.1597
Boiler[+]	0.6057	0.1573
Floor[++]	0.6016	0.1563
Water[++]	0.5997	0.1558
PC[+]	0.5909	0.1535
Gas/Electric Stove[+]	0.5894	0.1531
Mixer[+]	0.5580	0.1450
Vehicle (Car) [+]	0.5039	0.1309
TV[+]	0.4555	0.1183
Ceiling[++]	0.4484	0.1165
Audio system[+]	0.4443	0.1154
Electricity[+]	0.2685	0.0698
Vehicle (SUV) [+]	0.2543	0.0661

Determinant of the correlation matrix=0.086

Bartlett test of sphericity p-value=0.000

Kaiser-Meyer-Olkin Measure of Sampling Adequacy=0.876

Scale reliability coefficient (Cronbach's Alpha)=0.7738

[+]Variable is dichotomous indicating presence/absence of attribute

[++]Variable is categorical with more than two levels (i.e.: "Floor" refers to building materials inside the household's floor: 0=Dirt or no material; 1=Cement/Bricks; 3=Tile, wood, mosaic or other)
